# Tripping on the edge of consciousness

**DOI:** 10.1093/sleepadvances/zpad039

**Published:** 2023-11-06

**Authors:** James M Krueger

**Affiliations:** Integrative Physiology and Neuroscience, College of Veterinary Medicine, Washington State University, USA

**Keywords:** sleep, Factor S, cytokines, peptidoglycan, muramyl peptides, sleep function, local sleep

## Abstract

Herein the major accomplishments, trials and tribulations, and epiphanies experienced by James M. Krueger over the course of his career in sleep research are presented. They include the characterization of a) the supranormal EEG delta waves occurring during NREMS post sleep loss, b) Factor S as a muramyl peptide, c) the physiological roles of cytokines in sleep regulation, d) multiple other sleep regulatory substances, e) the dramatic changes in sleep over the course of infectious diseases, and f) sleep initiation within small neuronal/glial networks. The theory that the preservation of brain plasticity is the primordial sleep function is briefly discussed. These accomplishments resulted from collaborations with many outstanding scientists including James M. Krueger’s mentors (John Pappenheimer and Manfred Karnovsky) and collaborators later in life, including Charles Dinarello, Louis Chedid, Mark Opp, Ferenc Obal jr., Dave Rector, Ping Taishi, Linda Toth, Jeannine Majde, Levente Kapas, Eva Szentirmai, Jidong Fang, Chris Davis, Sandip Roy, Tetsuya Kushikata, Fabio Garcia-Garcia, Ilia Karatsoreos, Mark Zielinski, and Alok De, plus many students, e.g. Jeremy Alt, Kathryn Jewett, Erika English, and Victor Leyva-Grado.

## Introduction

Herein, I relate my delight in scientific discovery, the accompanying epiphanies, and the human intellectual interactions involved throughout my biomedical research career. I focus on just seven of my accomplishments. I emphasize the discoveries discussed involved scientists in addition to me.

Neuroscience is an unusual scientific field; we lack solid explanations of the origins of many important brain outputs, e.g. perception, thought, consciousness. In other words, we do not know what we are talking about in terms of brain emergent properties that provide evolutionarily fitness. Yet, we are a most sophisticated field with some of the smartest people in the world devoting their every waking minute to their research. Our knowledge of molecules and cells is massive, beyond the grasp of any one individual. We can predict pathological outcome in response to specific brain injury including loss of small numbers of specific brain cells. We are superb at manipulating aggregate behavior as evidenced by marketing successes, Muzak, subliminal advertising, and political campaigns. Yet, no one can explain how even the simplest percept emerges from the dazzling complexity of the molecular and cellular milieu so extensively characterized in our literature. To use a vernacular metaphor, we have described detailed features of the trees yet are unaware of how the forest got there. For the young inquisitive reader, this is an open invitation to a life’s work of challenging, interesting, and meaningful pursuits full of opportunity to address some of the questions puzzling mankind for eternity.

Sleep fits snugly into neuroscience; we debate what is its primordial function and exactly what it is that sleeps. This is preposterous; we spend a third of our lives asleep, Yes! about 27 years in total. We intuitively recognize the importance of sleep to our physical and mental performance and our health and well-being. We likely know someone with a sleep disorder and most of us have transiently experienced sleep disturbance. Within regulatory agencies, the costs and safety impact of disturbed sleep are well-known and very large. Deadly mistakes are made in the hospital, on the road, and elsewhere by the sleepy. Sleep scientists have ferreted out many discoveries relating molecules, cells, neuronal networks, and behavior to sleep. Yet, we cannot verify our grandmother’s advice to sleep well for prevention of and recuperation from infection and injury and for a fulfilling life. Nevertheless, within the last 50 years, sleep research has brought neuroscience to the cusp of explanation of many of these unknowns. This essay will inform the reader of some of these issues through a mixture of description, explanation, personal discovery, and my perception of the importance of a few of the advances I contributed.

## Excessive EEG Delta Wave Amplitudes Characterize Sleep After Sleep Deprivation

My first publication in sleep described the increases in electroencephalogram (EEG) slow wave (delta waves 0.5–4 Hz) amplitudes that occur during intense sleep immediately following sleep deprivation [1]. Our quantitative description of the enhancement of EEG delta waves post sleep loss has withstood the test of time. Our experimental hypothesis was that a sleep-promoting substance (Factor S) accumulated in brain during sleep deprivation and that when transferred to a control animal would induce excess sleep. While the induction of excess non-rapid eye movement sleep (NREMS) after the transfer of Factor S to recipient animals occurred and thereby helped validate our hypothesis, we also noticed an increase in amplitudes of EEG slow waves during NREMS post Factor S injections. This gave us pause because we did not know if it was an experimental artifact or due to the contamination of Factor S samples during the purification process. We were able to quantify the EEG delta wave amplitudes because John Pappenheimer had designed electronic hardware to band-pass filter the EEG signal retaining the 0.5–4Hz component and then calculate rectified root mean square voltages for corresponding time epochs used for manual scoring of sleep stages. Thus, after we noticed the NREMS EEG slow wave increases after Factor S injections, logically, if our hypothesis was correct, EEG delta wave amplitudes should also increase during the deep sleep following sleep deprivation. It did, and because we did not inject anything, we concluded that the sleep enhancing properties of Factor S, including enhanced EEG delta waves, were indeed due to Factor S. Thus, we used the enhanced post-sleep deprivation delta waves as an additional property of Factor S whether accumulating endogenously or injected after purification from another animal. This finding was of great importance to us because it gave us additional motive to continue our quest to purify Factor S. As it turned out, we needed a lot of motivation because it took six more years before structure was at hand [2, 3].

In retrospect, it now seems likely that if Alex Borbely had not periodically visited our Harvard laboratory, our findings may have been lost for years. Alex subsequently confirmed our findings. He went on to develop the use of Fast Fourier Transformation (FFT) analytical methods for EEG analyses and developed his two-process model of sleep. EEG slow wave power is a key variable used to quantify Process S in the two-process model. EEG delta power is often used as a measure of sleep intensity; however, that is a complex story [4].

## Factor S Is a Muramyl Peptide

In 1974, I joined John Pappenheimer’s laboratory as a postdoctoral fellow. He recruited me to isolate Factor S, a substance that accumulates in cerebrospinal fluid during wakefulness and induces sleep. At that time. neuropeptides were hot; it seemed new ones were being discovered almost every week. There was an unstated hope, based on the success of the endocrinologists, that there would be a “sleep hormone,” a substance whose actions were specific to sleep and caused sleep. When I joined the laboratory, John and his long-term collaborator, Manfred Karnovsky, had described Factor S as a low-molecular weight peptide and determined its elution profile on gel filtration. However, there was much that they did not know, including the molar amounts needed to induce sleep. My job, in theory, was simple. Use chromatography to fractionate the sample, test each subsequent fraction for sleep-promoting activity, and then keep on repeating this cycle using the fractions enriched in sleep-promoting activity at each step, but a different chromatographic technique, until the substance was pure. Once pure, we would do an amino acid analysis to determine composition.

Six years passed. No structure. No publications except the one mentioned above. Much bad news. At one point, a technician dropped a frozen sample representing an entire year of work; she watched it melt on the floor then wiped it up with a paper towel! NIH cut off our funding in the middle of the award project period, my salary was paid from that grant. John went to the Dean to ask for my salary to no avail. A year or so later, NIH turned down a separate grant I submitted; they had six people do a site visit to our lab. The visitors took a dim view of what we were doing and concluded that sleep was not regulated by chemicals but by neurons (the old soup and spark argument had resurfaced). Ironically, the cost of the site visit was about the same as I had requested in my grant. Also, one of the NIH review team asked during the site visit for a sample to test in their lab! This ethical blunder occurred during the review of my first NIH application.

I was within weeks of being unemployed. I had interviewed for faculty positions; the response was always the same—get Factor S structure, then come back. The thought of not being at the forefront of science was rather depressing yet for a few weeks it seemed as if that might be my fate. Just before my salary was to stop, the Office of Naval Research (ONR) saved our research. John had helped organize the ONR during WWII and some of his friends were still there. ONR awarded temporary emergency funding to us.

Those six years were not wasted (ONR was made aware of this). We made progress. We knew that we were on the edge of Factor S structure. We had scaled-up to extract large amounts of brain and urine. We had, with the help of Merck Co. and later Sterling Winthrop Co., extracted Factor S from 15 000 brains obtained from sleep-deprived rabbits, and in a separate preparation, 5000 L of urine obtained mainly from medical students. We knew that Factor S was very potent; for instance, after purifying Factor S from the 15 000 brains, we could not see anything in the test tube, yet there was enough Factor S there to put thousands of rabbits to sleep! Every week we were making the progress. Despite our financial woes, our samples were becoming clean enough that we could begin to discern amino acids upon analysis. Finally, success. On a clear fall morning, I climbed the two flights of stairs to where our amino acid analysis instrument was located. There waiting for me were the results from hydrolyzed and unhydrolyzed samples we had loaded the evening before. I quickly made some ballpark estimates of the amounts of the amino acids that were present, and realized we had good molar ratios. There was one peak present that was not a normal constituent of mammalian tissue; I had to consult the chromatography texts in the room to identify it as diaminopimelic acid. It was in equal molar ratio to the other amino acids indicating that it was part of the molecule we were after. It was the next few minutes of my life that I will never forget.

I knew what diaminopimelic acid was; it is the precursor to lysine. Humans do not have a D-amino acid decarboxylase, thus we cannot make lysine from meso-diaminopimelic acid and that is why lysine is a dietary essential amino acid. I knew that diaminopimelic acid was a component of bacterial cell walls; I knew that because Manfred Karnovsky’s lab mainly studied macrophages and how they were stimulated by bacteria to produce superoxide. I had sat through many of his lab meetings and had learned of bacterial cell wall structures, my mind had thus been prepared. After identifying diaminopimelic acid, I started a slow walk down the stairs back to my office. My mind was racing. My first thought was, maybe this is why one feels sleepy when sick. Other implications of the finding began to sink in. Mammalian cells were using bacterial products to signal. Did our finding represent an example of endosymbiosis? (At that time, I was unaware of Lynn Margulis’ endosymbiont hypotheses, so this thought was more troubling than it should have been.) Was our new evidence suggesting that humans are also holobionts? About halfway down the stairs, I made the mental link to cytokines, which at the time were very new. I knew from Manfred’s work that bacterial products were inducing production of cytokines in macrophages. They were not thought to be produced in the brain let alone involved in sleep regulation (it would take a few years before we showed that). Yet, I had the gut feeling at the time that this innate host defense mechanism may also work in the brain but for another purpose. These thoughts were radical, out of the box. The implications I knew would be rejected by neurobiologists, and especially by philosophers, as it was hard to avoid the thought that bacteria were influencing consciousness. When I suggested this within a physiological framework, I literally was booed at scientific meetings, despite the fact it was well known at the time that some bacterial infections were associated with coma. The fuzzy line between physiology and pathology was, and remains, too arbitrary for neurobiologists. The epiphanies I enjoyed that morning led to many new avenues of research. Regardless, I remain melancholic because what I considered the most important part of those thoughts, the link between microbes and mind, has remained taboo. Nevertheless, I am convinced that if anyone ever cites my work 100 years from now, it will be for those thoughts which crystallized that morning.

In 1982, we published the amino acid composition of Factor S [2] and concluded that it was very close to, if not part of, structures found within bacterial cell wall peptidoglycan. In the last paragraph of that publication, we speculated that mammals were using bacterial products for sleep regulation, like other molecules obtained from bacteria that are used in mammalian physiology. That paper led to many different directions in sleep research ranging from: (1) mass spectrometric characterization of our sleep promoting substance [3], (2) cytokines in sleep regulation [5, 6], (3) muramyl peptide structure—somnogenic properties [7], (4) characterization of other bacterial cell wall products in sleep, e.g. lipopolysaccharide [8], investigation of cell walls of archaebacteria which at the time were not known to be present in humans [9], and (5) determination of the effects of a variety of infectious agents on sleep including virus, protozoan, and fungi [10–13]. Muramyl dipeptide (MDP) is the minimal structure within Freund’s complete adjuvant retaining immune adjuvant activity, that finding led the way to investigation of the somnogenic properties of cytokines because MDP and other muramyl peptides induced cytokine production. Interestingly, Shai Shoham, an Israeli postdoc in my lab, showed that MDP-induced robust NREMS responses in insomniac rabbits with large anterior hypothalamic lesions suggesting muramyl peptides promote sleep via mechanisms independent of the hypothalamus [14]. Years after our identification of Factor S as a muramyl peptide, we cloned a peptidoglycan-binding protein (now called Pglyrp1) and showed its mRNA increased in brain during sleep deprivation [15]. The current activity of my lab is focused on quantification of peptidoglycan/muramyl peptides in brain and how and where they vary with physiology including sleep and time of day [16].

## Cytokines Are Well Characterized for Their Role in Sleep Regulation in Health and Disease

Our report of the somnogenic actions of muramyl peptides drew the attention of Louis Chedid, Institute Pasteur, and Charles Dinarello and Sheldon Wolff at Tuffs University. They were leading figures in immunology and medicine and in the new cytokine field, and acquaintances of John and Manfred. This was in 1981; Charles had already reported characteristics of endogenous pyrogen (later renamed interleukin-1 β [IL1]) but had not yet determined IL1 structure. I was moving to my first tenure-track academic position at Rosalind Franklin University. After multiple discussions, it was decided that I should take the IL1 project with me because John was close to retirement.

Charles offered purified IL1 samples to us, and John picked them up at Tufts using his bicycle as transport and later mailed them to me in Chicago. By 1983, Charles, Louis, and I had published an abstract [5] describing the somnogenic properties of IL1 followed by the full-length manuscript in 1984 [6]. This was a critical finding for my career for many reasons: (1) the scientists, with whom I was keeping company with (Pappenheimer, Karnovsky, Dinarello, Chedid, Egar Lederer, Klaus Biemann, Shozo Kotani) were superstars; (2) it was easier to get NIH funding for cytokines promoting sleep than for bacterial cell wall products doing such, due to NIH’s super-conservative review system; (3) the cytokine field rapidly expanded in depth and breadth; (4) the new field of psychoneuroimmunology had just begun.

Cytokines, well-known for their host defense roles, likely evolved for other purposes over 500 million years ago [17, 18]. Thus, the mammalian cytokine, epidermal growth factor (EGF), increases inactivity (aka, sleep) in *Caenorhabditis elegans* [19] and NREMS in mammals [20]. Tumor necrosis factor α (TNF) is antimicrobial and a mammalian sleep regulatory cytokine [21]. TNF’s *Drosophila melanogaster* homolog, EIGER, and IL1’s *Drosophila* analog, SPATZEL, affect fly rest/activity (aka-sleep) [22, 23]. Because cytokines, e.g. TNF, are key to host anti-microbial defenses and inflammatory responses, and antimicrobial TNF and peptidoglycan recognition protein 1 (PGLYRP1) in mice [24] and *nemuri* in *Drosophila* [25] are linked to mammalian sleep and *fly* locomotor activity, respectively. These observations suggest that microbial molecular signals integrated into primordial brain activity regulation and later into the mammalian brain cytokine sleep regulatory network. Furthermore, cytokine downstream mechanisms are shared by mammals and invertebrates, e.g. CREB-binding protein [26], nuclear factor kappa B (NFkB) [27, 28], ATP [29], and adenosine [30]. These effector molecules are produced on-demand in response to cellular activity, e.g. neuron action potentials and cell damage, and are initiated within local circuits [31]. I emphasize that these molecules are also involved with the emergence and integration of sleep with other activity-initiated local circuit phenomena, brain plasticity, inflammation, metabolism, and cerebral blood flow. The regulations of these brain processes are, like sleep, initiated within localized areas and are posited sleep functions [31]. Cytokines also made possible the defining of molecular causative pathways between microbes and host sleep [32–34]. Thus, not only are the mechanisms for local sleep inseparable from the connectivity/metabolic function of sleep, but they also cause the network outputs that lead to the necessity for the altered consciousness that typically pervades sleep.

Multiple cytokines affect mammalian sleep [6, 21, 35–38]. The somnogenic actions can be complex for individual cytokines; by way of example, I focus on IL1 and TNF in this section. Low-IL1 doses increase both the duration of NREMS and EEG slow-wave activity during NREMS during the day. Higher doses of IL-l have divergent effects on NREMS duration and EEG slow-wave activity, and the direction of the changes depend on the diurnal cycle [39]. This contribution, of which Mark Opp is the lead author, clearly demonstrated that the actions of IL1 on different sleep phenotypes are independent of each other and depend on the time of day. Subsequently, as an independent investigator, Opp contributed many additional cytokine/sleep publications [40]. He also went on to become the president of the Sleep Research Society and the PsychoNeuroImmunology Research Society. IL1 requires an IL1 receptor accessory protein (called AcP) to signal. AcP has three isoforms of which AcP is found in most cells and a neuron-specific AcP (called AcPb) found only in brain. Wild-type mice upregulate brain AcPb mRNA during sleep deprivation, but not AcP mRNA [41]. AcPb null mice lack sleep rebound post sleep loss and have less spontaneous sleep suggesting that the sleep actions of IL1 depend upon brain/neuron IL1 [42].

**Figure 1.**
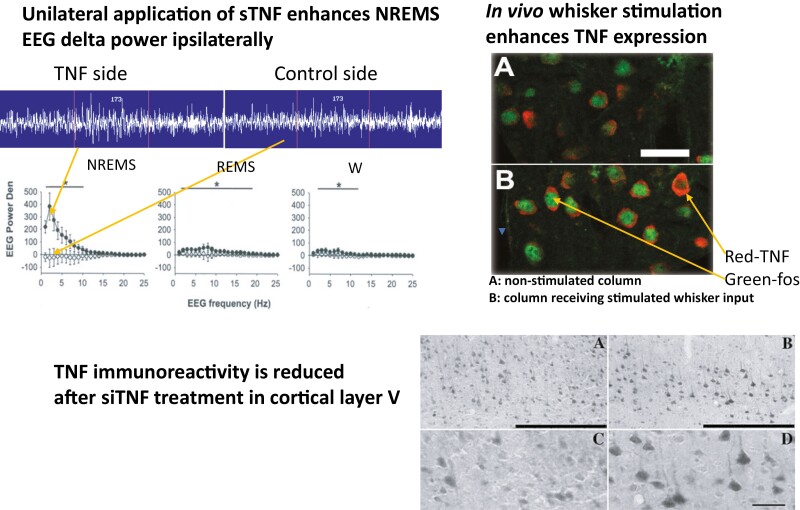
TNF enhances local NREMS EEG slow wave power, left figure [45]. Cortical neuronal TNF expression is enhanced by increased afferent stimulation, right figure [46] and is decreased if TNF expression is reduced using a TNF siRNA, lower figure [49]. After TNFsiRNA is given ipsilaterally, EEG delta power is reduced for a few days on the TNF siRNA-treated side but not on the control side [49].

In a different study [43], we showed that optogenetic stimulation of neurons upregulated IL1 and TNF protein expression in cultures of somatosensory cortical neurons. Furthermore, local unilateral applications of either IL1 [44], TNF [45, 46], or GHRH [47, 48] onto the surface of the cortex promote EEG delta power during NREMS ipsilaterally but not on the contralateral side. If, in parallel experiments, TNF (Figure 1) [49] or IL1 [44] or GHRH [47] is inhibited, then EEG delta power is inhibited ipsilaterally. These results suggest that these substances are involved in regulating local physiological EEG delta power and suggest the downstream effector mechanisms are localized as well (see below).

Another example of the complexity of cytokine actions on sleep involves TNF [21]. TNF is produced as a transmembrane (tm) 26 kD molecule. The extracellular moiety of tmTNF is cleaved by TNF converting enzyme (TACE) releasing 17 kD soluble (s) TNF into the extracellular space. Similarly, tmTNF receptors are hydrolyzed by TACE to release soluble (s) TNF receptors. sTNF via canonical forward signaling promotes sleep whereas sTNF receptors interact with tmTNF and inhibit sleep [50, 51]. This latter action is called reverse signaling. TNF is also well-characterized for its involvement in synaptic scaling (discussed below) and in glutamatergic AMAP Ca^++^ signaling [52–54].

Many years after our first description of IL1 and TNF somnogenic activity, neurobiologists finally acknowledged physiological roles for IL1 and TNF including sleep regulation [55]. The cytokine sleep literature is now very large and has helped clarify several important issues in sleep research that laid dormant for years. Thus, the concept of “local sleep” grew out of the cytokine-sleep literature from our knowledge of local production of cytokines involved in inflammatory responses and by addressing the question; “What is the minimum neuronal/glia network capable of sleep?” [33, 34, 56]. Because cytokines are pleiotropic and initiate responses within small groups of cells, they provide molecular links to sleep functions, e.g. metabolism, cerebral blood flow, inflammation, and plasticity. And that led to the idea of sleep being part of symbiotic host—microbiome processes [2, 57, 58].

## Cytokine Sleep Mechanisms Led to the Identification of Multiple Sleep Regulatory Substances

During our cytokine and microbe sleep work, we investigated multiple other SRSs; herein I focus on only a few of them. The people involved included Ferenc Obal Jr, Levente Kapas, Eva Szentirmai, Alok De, Jeremy Alt, and Ping Taishi. Obal and Taishi were long-term collaborators of mine, De a postdoctoral fellow, and Kapas, Szentirmai, initially graduate students of Obal’s in Hungary then became postdoctoral fellows in my lab, and Alt was a graduate student in my lab.

Obal had implicated growth hormone releasing hormone (GHRH) in experimental animal sleep regulation from his University of Szeged, Hungary laboratory. He had written to my former mentor Pappenheimer, who by that time had closed his experimental lab, and John in turn recommended that he write to me. We subsequently met each other at a scientific meeting in Puerto Rico in 1986. We immediately became interested in each other’s research and agreed to plan for Ferenc to work in my laboratory for 3 years. It was one of the best decisions of my life, as Ferenc was a scientist’s scientist; academic, soft spoken, diplomatic, hardworking, and very bright. We worked on many projects, but here I only relate what we did with GHRH and IL1. We showed that mice lacking a functional GHRH receptor (called *lit/lit* mice because they were little) had low spontaneous sleep [59]. Furthermore, we showed that anti-GHRH antibodies could block IL1-induced sleep responses thereby suggesting that GHRH was downstream from IL1 in terms of sleep promotion [60]. Later, in collaboration with Alok De, we showed that GABAergic anterior hypothalamic neurons increase their intracellular Ca^++^ concentration whether stimulated with either GHRH or IL1 suggesting a common cell is mediating their sleep responses [61]. From there, Jeremy Alt, joined this work and showed that *lit/lit* mice upon influenza challenge ([62]; see next section) instead of sleeping more for several days, *lit/lit* mice sleep less and displaying an unusual state of high-electromyograph tone and high-EEG slow-wave activity that we could not classify as either sleep or waking. Regardless, the infected *lit/lit* mice displayed progressive decreases in sleep and died rapidly from the virus infection. We even chronically infused growth hormone because GH is involved in host responses to infections, but it did not alter the downward spiral leading to the death of *lit/lit* mice. To date, this is the most dramatic example of changes in sleep responses to viruses that we have characterized. It clearly implicated both IL1 and GHRH in critical sleep regulatory responses during microbial challenge.

In separate studies, Eva Szentirmai and Levente Kapas had shown that GHRELIN plays a role in sleep regulation. Sleep, feeding, metabolic, and thermoregulatory mechanisms overlap in the hypothalamus. In mice, low-ambient temperatures and food restriction induce torpor bouts of body temperature reductions of the order of 5°C and characteristic metabolic and sleep changes. For this work, we obtained mice lacking the preproghrelin gene. In response to fasting at 17°C, preproghrelin null mice enter hypothermic bouts associated with reduced sleep, culminating in a dramatic drop in body temperature to near-ambient temperature levels. As the mice reached 25°C, the EEG went flat, but the dramatic hypothermia continued to drop. This hibernation-like state spontaneously reversed upon increasing ambient temperature to normal values, and the mice survived [63]. GHRELIN and IL1 share nuclear factor kappa B signaling mechanisms (also shown by us to be involved in sleep regulation [27, 28]); both IL1 and GHRH are involved in inflammation, and they affect each other’s expressions. Regardless, the sleep responses to lower ambient temperature and food restriction are the most dramatic we have thus far dealt with [64].

Based upon the knowledge that TNF, IL1, and other cytokines signaled in part via nitric oxide, Kapas *et al.* [65–67] showed that NO promotes sleep while its inhibition reduces sleep duration. This was one of our early cytokine-sleep downstream mechanisms that we investigated. That said, we have yet to determine if the IL1/GHRH sensitive GABAergic hypothalamic cells described above also require NO for somnogenic activity. As our NO developed, Kapas obtained NIH funding for the work which he pursued at Fordham University.

## Sleep Changes Dramatically During Infectious Diseases

At the time, we characterized Factor S as a muramyl peptide, I was unaware that sleep had not been measured over the course of an infectious disease. I just assumed that it was well-documented that sleep was enhanced during recuperation and served a helpful purpose. After a few academic seminars where I asserted this assumption and being unable to provide the documentation for such claims, I changed my tune. I started asking the audiences if they knew of any evidence pertaining to sleep responses during infectious diseases; none had. After one of my lectures, Linda Toth approached me. Linda had just arrived at the University of Tennessee with a background in pharmacology (PhD), Veterinary Medicine (DVM), and lab animal veterinary science. She indicated she could document sleep changes over the course of an infectious disease; something Hippocrates indicated happened but something that had not been measured over the next 2400 years. Linda and I joined forces; the research plan was simple at first, she would be given our EEG- and thermistor-equipped rabbits for infectious disease work. Our first efforts focused on *Staphylococcus aureus*. She injected live *S. aureus* intravenously to mimic a septicemic condition. Then the EEG and body temperature were recorded for multiple days. The results were in part surprising to us. On the first post injection day, after a few bacterial replication times, sleep increased as did brain temperature. However, by the second day after infectious challenge, sleep fell to below control recording values taken before infectious challenge and remained low until we sacrificed the animals. Also surprising was that by the second day, the fever persisted but not excess sleep [10]. This convincingly demonstrated that fever and sleep responses do not drive each other.

Linda and I continued to collaborate for several years to investigate sleep responses to both Gram-negative and other Gram-positive bacteria, fungi, and other infectious agents. Although in each experiment sleep responses occurred, the specifics of their timing and magnitude varied with infectious species and with the infection location. Regardless, the broad conclusion was what many had already experienced that sleep, and sleepiness, often associate with bouts of infection. Of great importance, using a *Trypanosoma brucei brucei* septicemia infectious model in rabbits (sleeping sickness), Linda showed that when *T. brucei* underwent antigenic shifts (its defense mechanism occurring every 21 days in rabbits), a new immune response was initiated by the host which was accompanied by enhanced sleep. This result clearly linked sleep to ongoing immune and accompanying inflammatory responses [68]. Years later, this has become an issue in multiple modern experimental manipulations where genes are introduced to specific cells and locations in brain using injected viral vectors; the injection-induced cellular damage, viral envelop-induced inflammations, and the substance in the viral vector likely interact with sleep altered inflammation rendering interpretation of results problematic.

Linda and I also addressed the issue of whether sleep during infections served a protective function [11]. One might initially think that all one needs do is to determine what happens to microbe-induced sleep responses in sleep-deprived animals. However, it is very difficult to isolate sleep as an independent variable, as during sleep almost every physiological bodily function changes thus there are many things to control for, e.g. stress responses, hypercapnia, hormone changes, appetite, multiple behaviors, inflammation, cognition etc. Regardless, we found that in rabbits that had robust sleep responses within the first 12 hours following infectious challenge they survived the infection, whereas as those animals that did not sleep more during the 12-hour period had a much worse prognosis and failed to survive the infection. While this might reinforce one’s grandmothers’ advice to get sleep to prevent and recuperate faster from an infection, the results are strictly correlative.

During our bacteria work (funded in part by the US Army Research and Development command), Dr. Jeannine Majde, who was a program officer with ONR and a virologist, contacted us because she was aware of our ONR-funded work (from a different division of ONR than Jeannine was associated with). (To avoid any hint of impropriety, we applied for NIH support for this work and got it). Jeannine introduced us to influenza virus and to a mouse adapted strain of influenza virus (one that passed about 600 times through mice and was not known to be infectious to humans). She initially suggested that we try infecting our rabbits with large doses of the mouse adapted virus to determine if sleep was altered; it increased [69]. However, this was an abortive virus infection model thus we rapidly switched to using mice.

When Linda left the University of Tennessee-Memphis, Linda and I developed our virus/sleep work independently. The work in my lab focused on finding what viral product was causing the sleep responses, where it was produced, and if it entered brain. Influenza virus is a single-stranded negative sense RNA virus. When it infects an animal, it will produce the positive-sense RNA strand, and it will anneal with the negative strand to produce double stranded (ds) RNA. Thus, our initial experiment was to show that intranasal administered influenza virus located to lungs and produced influenza dsRNA, which when isolated induced sleep responses in normal mice [70]. We also showed that intranasal challenge of mice with the mouse-adapted influenza virus caused robust sleep responses lasting several days. We went on from there to show that synthetic ds poly I:C [71], but not poly I or poly C, also induced robust sleep responses lasting about 24 hours. Then, we synthesized single-stranded viral positive sense and negative sense 108-mers and 661-mers and showed that the single stranded RNAs were inactive, but if annealed together both ds108- and ds661-mers induced sleep responses thereby confirming that short viral RNA strands could initiate sleep responses and that they needed to be double stranded [72]. In a later project, Victor Leyva-Grado, DVM joined our team and for his PhD thesis showed that within just a few hours of intranasal live viral challenge, influenza antigen locates to the olfactory bulbs of challenged mice. He went on to show that cytokines TNF and IL1 upregulate in olfactory bulb and hypothalamic neurons after intranasal viral challenge [73, 74], reviewed [75].

## What Is It That sleeps? Sleep Is Initiated Within Small Local Networks

In the mid 1980s–mid 1990s, I was busy building my career. Yet, my radical thoughts from my scientific youth never left; I questioned the dominant paradigm of sleep research. That being that sleep is a whole brain phenomenon regulated by a sleep-specific set of circuits (as mentioned above Shai Shoham and me [14] had already shown that insomniac hypothalamic-lesioned rabbits have robust sleep responses if given MDP; thereby raising doubt about the necessity of any sleep regulatory center). I kept asking unanswered questions seldom addressed by sleep researchers. What is the minimal component of brain capable of sleeping? When did sleep start in evolution? Why do we sleep? Is there a connection between sleep mechanisms and sleep function? (There need not be; think about circadian rhythms). Eventually, Ferenc and I had discussions about these issues and compiled a list of evidence that was eating away at the dominant sleep regulatory paradigm. For instance, Mukhametov [76] showed that dolphins sleep only on one side of the brain at a time. *Countless lesion studies led to the simple conclusion that no matter what area of brain was lesioned, if the animal or person survived the brain damage, it slept.* This strongly indicated that no circuit in brain was necessary for sleep; an unwanted concept to those studying sleep circuits. Mahowald and Schenck [77] described the simultaneous occurrence of different states in neurology patients suggesting that part of the brain could be asleep while other parts are awake, e.g. sleepwalking, sleep inertia.

Unlike my fall 1980 epiphanies following Factor S sequence determination, this was a slow, hard process. It required much academic discipline because we knew that we were heading for a new theory of brain organization of sleep and that it would challenge the sleep research community. Finally, we agreed to publish a version of our theory [56]. Its main tenets were that sleep was a property of, and initiated in, small groups of highly interconnected neurons. A second tenet of the theory was that, depending upon the intensity and patterns of neuronal network use during wakefulness, sleep is initiated in, and thus targeted to, small networks in brain. Third, we expanded the scope of the theory to include sleep function (see next section) proposing that sleep served to preserve brain plasticity. These ideas provided a unique way to frame sleep mechanisms and function. Within the year, Lee Kavanau of UCLA [78], came to similar conclusions using independent logic. He also proposed that sleep was required to preserve plasticity and called sleep a dynamic stabilization state. Second, Borbely and colleagues tested our theory [79]. They showed that excessive unilateral stimulation of the somatosensory cortex in humans using a vibrator held in one hand enhanced the EEG delta wave power in the contralateral cortex during the first subsequent NREMS epoch. Their results were consistent with our theory, sleep is targeted to areas of brain depending upon use during prior wakefulness. Their results have been replicated in different ways many times by others in the intervening years (see next paragraph). In addition, Tononi and Cirelli [80] proposed their synaptic homeostasis hypothesis positing that glutamatergic neurons downregulate during sleep to stabilize glutamatergic synaptic networks. In our view, their theory is too narrow a version of ours and Kavanau’s theories by focusing mostly on glutamatergic transmission. That said, it has had great appeal in its simplicity and its focus on cortical mechanisms. However, it does not adequately address memory literature suggesting that sleep is required to preserve newly formed synapses [81–83].

Within the past few years our view of brain organization as it applies to sleep has become even more acceptable. Modern imaging studies have shown that there is differential activation of the brain during sleep that is dependent upon what part of the brain is used during wakefulness [84, 85]. Electrophysiological studies have shown that neurons within a visual cortex receptive field shift to a quiet mode in an organized manner as an animal is going to sleep while still performing a task [86]. Furthermore, cortical columns have different state properties depending upon prior activity and show sleep homeostasis following periods when an individual column is in a wake-like state for long periods [46, 87]. Waking activity and sleep differentially affect expression of molecules, e.g. homer, associated with synaptic plasticity depending upon the synaptic dynamics of the cortical column involved [88, 89]. Furthermore, the local sleep hypothesis predicts that any viable neuronal/glial network will oscillate between states; others and us have shown that mature in vitro cultures of cortical cells manifest a sleep-like default state [43, 90–92] and the sleep-like state is enhanced after IL1 [93] or TNF treatment [43] and inhibited by excitatory amino acids [91]. Our view of local sleep has thus modified the canonical sleep regulatory paradigm by inclusion of the initiation of sleep within small networks and their spontaneous state synchronization [94] and by the established sleep regulatory circuits, e.g. anterior hypothalamus. However, sleep regulatory circuits are not necessary for organism sleep or for the somnogenic actions of muramyl dipeptide [14]. We are thus left with a lot to learn.

## The Preservation of Brain Plasticity Is a Key Sleep Function

In 1993, we proposed a sleep function theory [56]. None of the prior theories seemed sound to us (including my own that sleep served an immune function) [57], because all the proposed functions could be achieved without an animal losing consciousness. Regardless, mammalian sleep functions seem clear at the whole animal level, e.g. calories are saved, performance is restored, and human affect becomes more positive; there is universal acknowledgement that sleep has multiple functions. As does, for example, respiration. During sleep, one is subject to predation and gives up opportunities to reproduce, eat, drink, or socialize. It seems likely that sleep could only have evolved, despite these high-evolutionary costs if it serves a critical function. Maintaining adaptable flexible neural connectivity may be sufficiently important to the brain to allow the persistence of such a periodic disadvantaged state [33]. However, at mechanistic levels, there remains a lack of consensus as to exactly what is affected, and the direction of the effects [83].

A central idea in connectivity theories of sleep function is the recognition that use-dependent changes in synaptic efficacy and connectivity would lead to dysfunction unless there were processes to stabilize, and thus preserve, functionally optimized synaptic networks (which are prima facie adaptive because the organism is alive). Regardless, brain activity is constant and functional circuits are constantly being modified by the use-dependent-driven changes in connectivity [56, 78]. Donald Hebb’s synaptic hypotheses posited that neuronal synaptic anatomy and transmission efficacy are dependent upon synaptic use, where synaptic use increases synaptic efficacy and disuse weakens synapses [95]. This is often referred to the “use it or lose it” hypothesis. (Parallels manifest elsewhere in physiology, e.g. muscle strength increases with use and atrophies if not used. Similarly, immunity in germ-free animals is greatly impaired compared to animals exposed to bacteria throughout their life.) Yet, a flexible brain that can remember and respond to changing stimuli is necessary for evolutionary fitness and survival. Thus, building on Edelman’s neuronal group selection theory [96], we [56] proposed that sleep is an evolutionary essential process for the preservation of synaptic plasticity, i.e. to counter excessive synaptic rigidity or atrophy—induced by disproportionately used, or unused, synaptic networks. Such preservation would provide for continued adaptation to an ever-changing environment [56].

**Figure 2.**
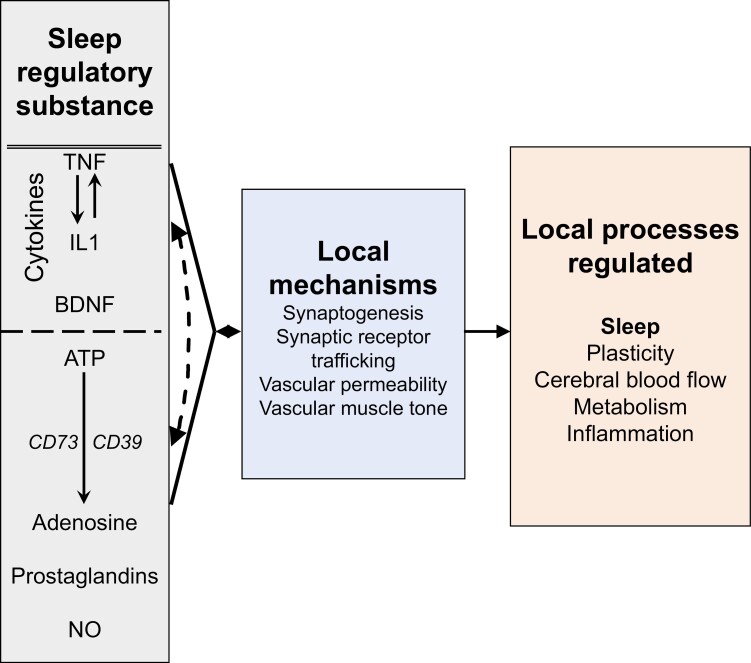
Sleep regulatory substances are produced within small networks in response to cell damage, sleep loss, or cell activity. Those substances affect a variety of synaptic mechanisms which, in turn, regulate multiple local processes. The processes shown are posited to be sleep functions [83].

However, without additional regulation of Hebbian plasticity, synapses become unstable, and synaptic diversity is lost [54, 89, 97, 98]. One example of how synapses change with use is called synaptic scaling. Synaptic scaling is a long-term (days) process. Synaptic scaling can operate in both excitatory (e.g. glutamatergic) or inhibitory (e.g. GABAergic) synapses. If use, e.g. action potential frequency/pattern is intense, receptors/ligand complexes traffic into the cell rendering the postsynaptic neuron transiently less sensitive to the extracellular ligand because access to free transmembrane receptors is reduced. In contrast, if few action potentials transmit across the synapse, the population of the ligand (neurotransmitter) membrane receptors increases and thereby increases the sensitivity of the postsynaptic cell to the transmitter. This is one of several neuronal plasticity mechanisms and it, like others, is involved in multiple brain processes including sleep regulation. A proposed mechanism for synaptic network stabilization involves SRS-induced changes in local electrical properties as described above (Figure 2). Indeed, there is now direct evidence that TNF, IL1, and brain-derived neurotrophic factor (BDNF) are involved in synaptic scaling and are upregulated in brain in response to cell activity. Kavanau [78] and Tononi and Cirelli [80] proposed that intrinsic spontaneous electrical activity (which would be altered by IL1, TNF, and BDNF produced in response to cell activity) serves this scaling function. Thus, not only are the mechanisms for local sleeping inseparable from the connectivity/metabolic function of sleep; they also cause the network outputs that lead to the necessity for the altered consciousness that typically pervades sleep [33].

Our theory that organism sleep emerges from multiple small networks does not yet address questions of how many small networks need to be in the sleep-like state before consciousness changes. Of note, a similar issue is present in the more traditional canonical paradigm of sleep regulation, which proposes a top–down imposition of sleep on the brain by regulatory circuits; it does not specify the areas (and how many of them) need to be acted upon by the regulatory circuits to produce sleep. Thus, although we are far from any comprehensive molecular or genetic understanding of sleep or the mechanistic details of local control of sleep, the present view continues to provide a new evolutionary conceptual structure.

## The Future of Sleep Research; An Opinion

*Within darkness, a freethinker will light a dim torch to enlighten the emergence of consciousness enabling it to discover itself before all life flickers out in anthropogenic heat.*

Without fulfilling this prophecy, there is no future for sleep research. Yet, it seems sleep research is needed and for that we should be thankful.
